# Treatment of Aortic Stenosis in Elderly Individuals in Brazil: How Long Can We Wait?

**DOI:** 10.36660/abc.2020003

**Published:** 2020-02

**Authors:** Marcelo Antônio Cartaxo Queiroga Lopes, Bruno Ramos Nascimento, Gláucia Maria Moraes de Oliveira

**Affiliations:** 1 Sociedade Brasileira de Cardiologia, Rio de Janeiro, RJ - Brazil; 2 Hospital Alberto Urquiza Wanderley, João Pessoa, PB - Brazil; 3 Universidade Federal de Minas Gerais, Belo Horizonte, MG - Brazil; 4 Universidade Federal do Rio de Janeiro, Rio de Janeiro, RJ - Brazil

**Keywords:** Life Expectancy, Aging, Aged, Stenosis Valve Aortic/surgery, Heart Valve Prosthesis, Heart Valve Prosthesis Implantation, Transcatheter Aortic Valve Replacement/trends

The growing life expectancy of the population is increasing the occurrence of diseases affecting the elderly age group, notably the non-rheumatic degenerative valvular diseases and, in particular, aortic stenosis, whose prevalence in elderly individuals older than 75 years has been estimated at 3 to 5%. This is the most common valvular heart disease among elderly individuals, and its severe form is associated with high morbidity and mortality. The life expectancy of patients with aortic stenosis presenting with heart failure and rhythm disturbances is estimated to be less than 2 years. The standard treatment for this disease is cardiac surgery with replacement of the aortic valve by a prosthesis. However, due to high surgical risk, especially in very elderly patients with associated comorbidities, cardiac surgery is contraindicated in about 30% of the cases or is performed with high morbidity and mortality rates according to preoperative scores. For these critically ill patients, a new, less invasive technique consisting of transcatheter aortic valve implantation (TAVI) of a bioprosthesis has been considered the therapeutic option of choice, initially tested in patients at very high surgical risk, but currently with evidence of noninferiority compared to open surgery in lower-risk individuals.^1,2^ The first TAVI procedure in the world was performed in France in 2002 by Professor Alain Cribier, and the method was pioneered in Brazil in 2008. Since then, a considerable number of patients has been treated. However, despite robust evidence of safety and efficacy, this therapy has still not been incorporated into the supplemental or public health care systems (*Sistema Único de Saúde* [SUS]) in the country.

Despite the fact that TAVI has already been evaluated and accepted in the health care systems of several countries with evidence of cost-effectiveness even in individuals at intermediate operative risk,^3^ the Ministry of Health, based on a position stand from the National Commission for Incorporation of Technologies in the SUS (Conitec), established in 2013, in response to a request by the Brazilian Society for Hemodynamics and of Interventional Cardiology (SBHCI) acting as the petitioner, considered that there was no convenience in incorporating this therapy in Brazil. Conitec, at the time, based on the opinion of a reviewer and disregarding the opinion of prestigious national universities presented in a public consultation on the subject, recommended against the incorporation of the procedure, justifying this decision on three points: a) TAVI would not be a safe and effective procedure due to an allegedly high incidence of stroke within the first 30 days after the procedure, b) the budgetary impact of incorporating TAVI into the SUS would approach 1 billion reais per year, and c) methodological inaccuracies could have affected the economic model presented by the petitioner and the PARTNER B study, which served as the main background for the incorporation of this procedure by all health technology assessment agencies in the world that have requested the incorporation of this technology.

Currently, robust data published since the issuance of this report, from at least six major randomized clinical trials and international registries,^1,2,4^ suggest no doubt about the appropriateness of the technique in selected patients, resulting in a recent expansion of risk groups in which TAVI would have similar results compared to open surgery, including a reduction in important outcomes like length of hospital stay and neurological events. However, there is no way to obscure the fact that the budgetary impact of the procedure can be high, especially given the demographic changes that the country has been going through in recent decades. Estimates of costs with the technique by the Ministry of Health, as discussed below, seem at first glance inaccurate and based on data that may not reflect the national reality. Estimates of the frequency of use of the procedure calculated by technicians of the Ministry of Health overestimate the access to TAVI in Brazil, projecting a budgetary impact that exceeds a billion dollars.

Data from the 2017 Global Burden of Disease (GBD) study analyzing non-rheumatic heart valve diseases show that even though the age-standardized prevalence of these grouped diseases has remained relatively stable in Brazil from 1990 to 2017, there was a significant increase in Non-rheumatic calcific aortic valve disease, from 53.5 (95% uncertainty interval [95%II]: 48.1 - 59.9) per 100,000 inhabitants in 1990 to 64.4 (95%II: 57.2 - 72.5) per 100,000 inhabitants in 2017 for both men (18.5%) and women (24.2%).^5^ The increase in the absolute prevalence rate of this valvular disease was even more significant, surpassing 114% (95%II: 105.5 - 124.3%) over 27 years, and suggesting a progressive and still growing impact from aortic valve diseases on the country's health care systems (Figure 1).^5,6^

**Figure 1:**
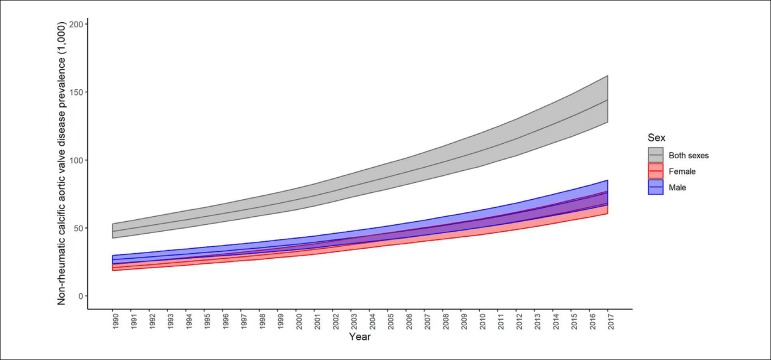
Absolute prevalence of Non-rheumatic calcific aortic valve disease in Brazil from 1990 to 2017 (Global Burden of Disease 2017).^7^

Regarding the causes of death in Brazil, non-rheumatic valvular diseases rose from the 10th position in 1990 to the 9th position in 2017. Although the age-standardized mortality associated with these valvular diseases has remained relatively stable, a considerable increase was observed for degenerative aortic valvular disease. The mortality rate at all ages due to non-rheumatic valvular diseases increased significantly by 87.5% (95%II: 63.5 - 96.9%) (Figure 2), with a large contribution from the population over the age of 70 years, especially in relation to Non-rheumatic calcific aortic valve disease, which showed a 108% increase in this age group in the evaluated period.^7^ These trends have also resulted in increased proportional mortality associated with Non-rheumatic calcific aortic valve disease in both sexes (Figure 3), suggesting a striking contribution from the changes in the age profile of the population in recent decades to the global burden of valvular disease in Brazil, with a notable impact by the aging of the population.^6^

**Figure 2:**
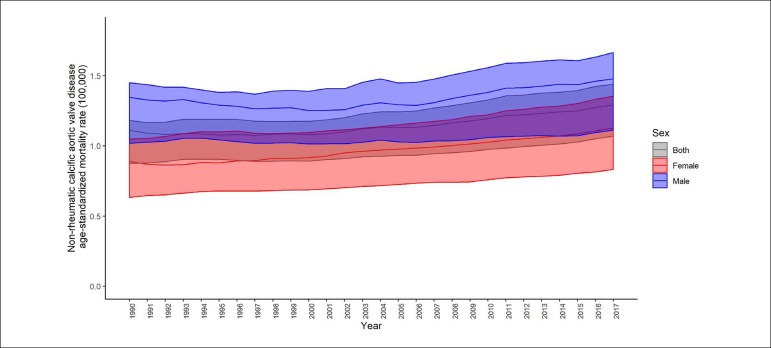
Mortality rate at all ages due to Non-rheumatic calcific aortic valve disease in Brazil from 1990 to 2017 (Global Burden of Disease 2017).^7^

**Figure 3:**
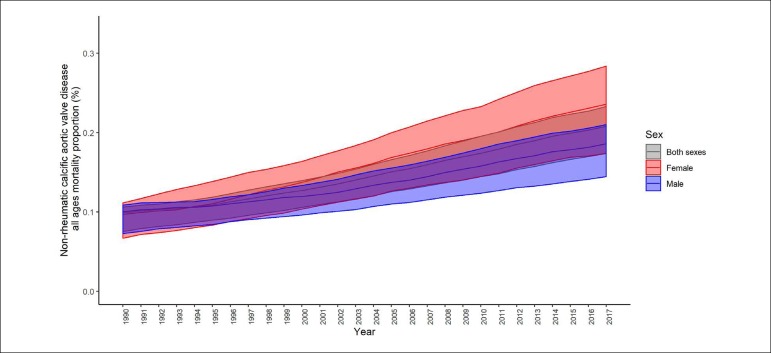
Proportional mortality from non-rheumatic calcific aortic valve disease in Brazil from 1990 to 2017 (Global Burden of Disease 2017).^7^

Additionally, GBD 2017 data suggest that socioeconomic development is also a determinant of some types of valvular diseases, notably Non-rheumatic calcific aortic valve disease: percentage changes in age-standardized mortality rates from 1990 to 2017 correlated significantly with the sociodemographic development index (SDI) of the Brazilian states in these years (1990: r^2^ = 0.17, p = 0.005; 2017: r^2^ = 0.23, p = 0.003) (Figure 4), and a similar pattern was observed for morbidity, with significant correlations between disability-adjusted life years (DALY) and the SDI in the period.^6^ These trends are in line with those observed for cardiovascular disease in general in Brazil and in other Portuguese-speaking countries.^8^ However, it should be noted that primary epidemiological data on degenerative aortic valvular disease in Brazil are still scarce, and most estimates derive from statistical modeling.

**Figure 4:**
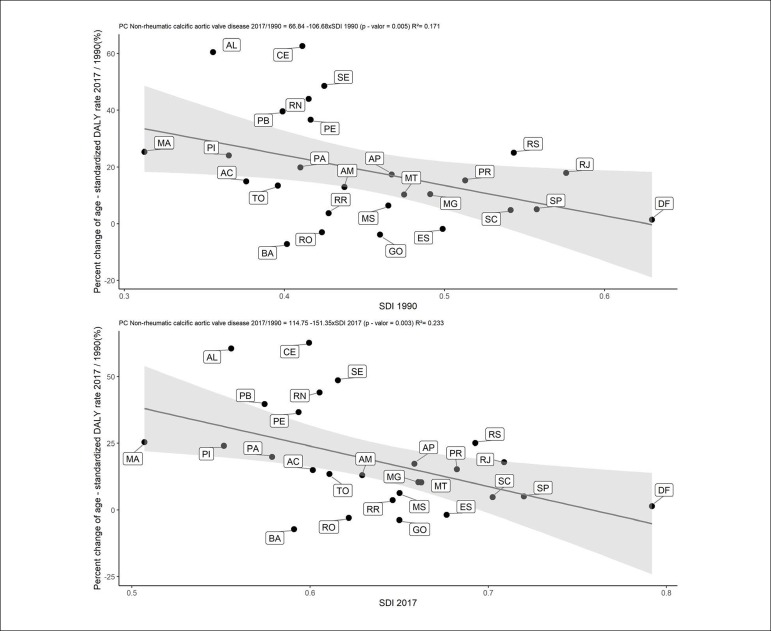
Correlation between the percentage change in mortality rates from non-rheumatic calcific aortic valve disease and the sociodemographic development index (SDI) by Brazilian state in 1990 (A) and 2017 (B).

Despite the progressive increase in the prevalence and disease burden associated with degenerative valvular diseases in the country, the number of annual hospitalizations by the SUS for treatment of valvular heart disease remained stable between 2008 and 2018, with a modest increase in costs of around 40%, not adjusted for the inflation in the period^9^ (Table 1). At the beginning of the period evaluated in this time series (2008), the first TAVI implantation was performed in Brazil, and current data from the RIBAC national registry, organized by the SBHCI, compute over 800 procedures, with rates of success and complications exceeding those in the literature.^10,11^ The approximate cost per transfemoral implantation is estimated at R$ 82,826.38, with the prosthesis corresponding to about 80% of this amount.^12^

**Table 1:** Number of hospitalizations related to the treatment of valve disease in Brazil from 2008 to 2018. Source: DATASUS.^9^

	2008	2009	2010	2011	2012	2013	2014	2015	2016	2017	2018	TOTAL
VALVE DISEASE*	1,606	1,753	1,647	1,878	1,938	1,938	1,849	1,940	2,068	2,023	2,090	20,730
VULVAR SURGERY	8,045	8,344	7,745	8,297	8,518	8,176	8,130	7,937	7,756	7,758	7,574	88,280
MITRAL VALVULOPLASTY	477	551	478	473	403	431	408	341	206	236	200	4,204
OTHER VALVULOPLASTY	451	477	445	486	456	527	515	513	399	427	391	5,087

* Hospitalizations due to other surgical procedures related to valve diseases

Conitec defended that the budgetary impact estimate was not the main determinant for its unfavorable opinion, but gave evidence contrary to this assertion when insisted on maintaining the estimate at levels completely dissonant from the reality of health care in Brazil, especially concerning the SUS. The new budgetary impact estimate was calculated after public consultation but was presented without emphasis in the final report (restricted to two lines on page 25), hindering its visibility, while the prior estimate modified by Conitec after the public consultation continued to be largely detailed on a table over several pages (for example, pages 17, 18, and 19), leading the reader to an inaccurate conclusion that this would still be the estimate that the commission considered to be correct.

The new estimate reduced the budgetary impact by more than 300 million reais per year. The Conitec considered that the budgetary impact would still be high, but did not indicate with clarity which levels were considered acceptable. Also unclear were the reasons why Conitec abandoned its first budgetary impact estimate without adopting the new estimate presented by the petitioner, which was based on the reality of health care in Brazil through its expert panel, indicating a far more feasible impact in view of the Ministry of Health budget and the provision of medical procedures to the Brazilian population by SUS. Instead, estimated data from other countries were primarily considered for the analysis.

Without a clear connection with the remainder of the opinion, page 25 of the final Conitec report presents the budgetary impact estimate, which is subject to several criticisms in addition to those presented. First, it should be emphasized the mentioning of the study by Wood^13^ showing the estimated increasing use of TAVI in Europe, with an annual rate of 40.9 million inhabitants, which would be proportional to 8,025 procedures/year in Brazil.

Conitec constantly criticizes the use of international instead of national data. Interestingly, in this case, to calculate the budgetary impact, Conitec used an European estimate, which would be particularly questionable in this context, since 1) TAVI has been registered and practiced for much longer in Europe than in Brazil; 2) the epidemiological characteristics of the European population are different from those of the Brazilian population in terms of being older and with a longer time elapsed since the epidemiological transition, which increases the number of patients aged over 75 years, and consequently the prevalence and the number of procedures. It is important to mention that the European population is estimated at 700 million inhabitants, that is, much larger than the Brazilian population; 3) the European health care systems, particularly in Germany, have a different stance from that of the Brazilian government regarding the emergence of technological innovations and is much more receptive to new health care technologies; 4) the Brazilian health care system, particularly specialized and medium/high complexity care, as is the case with TAVI, is not evenly distributed throughout the country, and besides, Brazilian hospital structure, diagnostic infrastructure, and clinical staff is not as widely available or efficient as those in European countries, and thus, the rate of diagnosis and procedures performed in the country would not be nearly compatible with European ones; 5) the number of 8,518 procedures/year is very close to the 9,000 procedures/year performed in the United States upon the request for incorporation (with the United States having a much larger population than Brazil), and, as mentioned in the public consultation, is a number incompatible with the epidemiological and health care access characteristics of the Brazilian population.

Still regarding the estimate of the number of procedures per year, it is noteworthy that, through a comparative analysis with similar procedures offered by the SUS, it is easily seen that such estimate is at least unrealistic. Table 1 shows, based on official data from the Department of Informatics of the Unified Health System (DATASUS), the number of surgical cardiac valvular procedures performed in Brazil between 2008 and 2018. It is noteworthy that the data provided by DATASUS do not allow the identification of the age of the patients, the valve treated, or the etiology and type of valvular disease. Data related to prosthetic valve implantation include both mitral and aortic implantations.

In this DATASUS estimate, the total number of surgeries for prosthetic valve implantation does not exceed 8,518 per year. In 11 years, 88,280 surgeries for prosthetic valve implantation were performed in Brazil considering all age groups and implantations of any heart valve. Additionally, the total number of valvular surgeries, including those associated with myocardial revascularization, multiple valvular replacement, and prosthetic valve implantation, reached a maximum of 11,315 procedures in the year of 2012. Because it is intended for a subgroup of older patients (> 75 years) with aortic stenosis, TAVI would certainly not have been performed with a frequency similar to that of all valvular surgeries performed in the SUS. Also, a large meta-analysis has suggested that among patients with aortic stenosis - even in developed countries - around 20% have a severe form of the disease. Of these, only about 60% would be eligible for TAVI, with 20% of inoperable or intermediate or high-risk patients. Of these, only about 60% would be eligible for TAVI, with 20% of inoperable or intermediate or high-risk patients - the better established indications for the procedure.^14^

Despite the absence of primary estimate data for TAVI indication in Brazil, we can apply demographic and epidemiological data for further detailing. Considering the latest GBD 2017 modeling in relation to the prevalence of calcific aortic valvular disease, we would have 64.4/100,000 inhabitants.^5^ Data from an IBGE census estimated an approximately 12.9 million elderly individuals aged ≥ 70 years in Brazil in 2019, which would result in roughly 8,400 patients with clinically significant disease in a very conservative estimate. If, alternatively, the estimated prevalence in the literature of about 3 to 5% in individuals ≥ 75 years is applied to IBGE data^15^ (a population of 7.7 million), and extrapolating the results from the largest available meta-analysis of aortic stenosis,^14^ we would have about 9,300 to 12,000 patients presumably eligible for TAVI in Brazil in 2019. Considering the estimates of the National Supplementary Health Agency that 24.3% of the Brazilian population had access to private health care plans in 2019, and also the aforementioned issues related to difficulties in access and infrastructure limitations in the annual history of valvular surgeries in the SUS,^9^ the projections of financial impact presented by Conitec are undoubtedly overestimated against objective data.

Of note, the Ministry of Health's technical position remains unchanged, at least in relation to the frequency of use and the budgetary impact of TAVI in Brazil. In the discussion of Project Act 5.460/2016 determining the mandatory coverage of TAVI in the SUS, which has already been approved by the Chamber of Deputies, the Ministry of Health - in response to the Finance and Taxation Commission on June 26, 2018, after 11 years from the request to Conitec - maintained its position regarding the projections discussed here.^16^

Finally, in addition to budgetary issues, it is of fundamental importance the development of technological infrastructure and professional training in the Brazilian public health system for the implementation of complex treatments in most diverse areas, in a process of industrial development of health care, contemporary to the inexorable process of medical innovation. Regarding the example of structural cardiovascular interventions, other modalities - such as percutaneous repair of mitral regurgitation and percutaneous treatment of congenital valvular heart disease - have already been described in the literature, and should soon be brought also to the discussion table of public policy managers and lawmakers. In this sense, emphasis should be placed on recent efforts by the national cardiological societies, with the seal of the Brazilian Medical Association, for the development of courses for training and professional certification.

In conclusion, the process of incorporating new technologies into the SUS - notably TAVI - must be currently discussed in depth and in a multidisciplinary and professional fashion, based on objective data and technical discretion, with due emphasis on epidemiological, technical, infrastructural, and budgetary issues.

The persistence of the overly restrictive position in the incorporation of technologies into the health care system in Brazil (in TAVI’s case, the delay in the incorporation exceeds a decade), in addition to resulting in the inconvenient phenomenon of requiring a judicial process, impels to other routes to obtain treatment access, which are much slower and more complex, such as the legislative path, thwarting the just expectations placed by the civil society in the Public Power, especially considering that universal, full, equal, and free access to the health care system has been established in Brazil in article 196 of the Federal Constitution.

## References

[1] Leon MB, Smith CR, Mack MJ, Makkar RR, Svensson LG, Kodali SK (2016). Transcatheter or Surgical Aortic-Valve Replacement in Intermediate-Risk Patients. N Engl J Med.

[2] Mack MJ, Leon MB, Thourani VH, Makkar R, Kodali SK, Russo M (2019). Transcatheter Aortic-Valve Replacement with a Balloon-Expandable Valve in Low-Risk Patients. N Engl J Med.

[3] Goodall G, Lamotte M, Ramos M, Maunoury F, Pejchalova B, de Pouvourville G (2019). Cost-effectiveness analysis of the SAPIEN 3 TAVI valve compared with surgery in intermediate-risk patients. J Med Econ.

[4] Zhou Y, Wang Y, Wu Y, Zhu J (2017). Transcatheter versus surgical aortic valve replacement in low to intermediate risk patients: A meta-analysis of randomized and observational studies. Int J Cardiol.

[5] Disease GBD, Injury I, Prevalence C (2018). Global, regional, and national incidence, prevalence, and years lived with disability for 354 diseases and injuries for 195 countries and territories, 1990-2017: a systematic analysis for the Global Burden of Disease Study 2017. Lancet.

[6] Institute for Health Metrics and Evaluation (2016). (IHME), Disease GB. GBD Compare | Viz Hub: 2018 University of Washington.

[7] Collaborators GBDCoD (2018). Global, regional, and national age-sex-specific mortality for 282 causes of death in 195 countries and territories, 1980-2017: a systematic analysis for the Global Burden of Disease Study 2017. Lancet.

[8] Nascimento BR, Brant LCC, Oliveira GMM, Malachias MVB, Reis GMA, Teixeira RA (2018). Cardiovascular Disease Epidemiology in Portuguese-Speaking Countries: data from the Global Burden of Disease, 1990 to 2016. Arq Bras Cardiol.

[9] Brasil, Ministério da Saúde (2018). Indicadores e Dados Básicos - Brasil.

[10] Nunes Filho ACB, Katz M, Campos CM, Carvalho LA, Siqueira DA, Tumelero RT (2019). Impact of Acute Kidney Injury on Short- and Long-term Outcomes After Transcatheter Aortic Valve Implantation. Rev Esp Cardiol (Engl Ed).

[11] Monteiro C, Ferrari ADL, Caramori PRA, Carvalho LAF, Siqueira DAA, Thiago L (2017). Permanent Pacing After Transcatheter Aortic Valve Implantation: Incidence, Predictors and Evolution of Left Ventricular Function. Arq Bras Cardiol.

[12] Bittar E, Castilho V (2017). The cost of transcatheter aortic valve implantation according to different access routes. Rev Esc Enferm USP.

[13] Wood S (2012). TAVI Numbers Rise in Europe as Reimbursement, Expertise Expands: Medscape.

[14] De Sciscio P, Brubert J, De Sciscio M, Serrani M, Stasiak J, Moggridge GD (2017). Quantifying the Shift Toward Transcatheter Aortic Valve Replacement in Low-Risk Patients: A Meta-Analysis. Circ Cardiovasc Qual Outcomes.

[15] Instituto Brasileiro de Geografia e Estatistica Cidades 2016.

[16] Brasil, Ministério da Saúde Parecer Técnico N.146-SEI/2017-DAET/CGAE/DAET/SAS/MS.

